# Frequent hemodialysis versus standard hemodialysis for people with kidney failure: Systematic review and meta-analysis of randomized controlled trials

**DOI:** 10.1371/journal.pone.0309773

**Published:** 2024-09-06

**Authors:** Patrizia Natale, Suetonia C. Green, Matthias Rose, Michiel L. Bots, Peter J. Blankestijn, Robin W. M. Vernooij, Karin Gerittsen, Mark Woodward, Carinna Hockham, Krister Cromm, Claudia Barth, Andrew Davenport, Jörgen Hegbrant, Pantelis Sarafidis, Partha Das, Christoph Wanner, Allan R. Nissenson, Benedicte Sautenet, Marietta Török, Giovanni Strippoli

**Affiliations:** 1 Sydney School of Public Health, The University of Sydney, Sydney, NSW, Australia; 2 Department of Precision and Regenerative Medicine and Ionian Area (DIMEPRE-J) University of Bari Aldo Moro, Bari, Italy; 3 Nephrology, Dialysis and Transplantation Unit, Department of Medical and Surgical Sciences, University of Foggia, Foggia, Italy; 4 Department of Medicine, University of Otago, Christchurch, Christchurch, New Zealand; 5 Department of Psychosomatic Medicine, Center for Patient-Centered Outcomes Research (CPCOR), Charité –Universitätsmedizin Berlin, Corporate Member of Freie Universität Berlin and Humboldt-Universität zu Berlin, Berlin, Germany; 6 Julius Center for Health Sciences and Primary Care, University Medical Center Utrecht, Utrecht University, Utrecht, The Netherlands; 7 Department of Nephrology and Hypertension, University Medical Center Utrecht, Utrecht, The Netherlands; 8 The George Institute for Global Health, University of New South Wales, Sydney, NSW, Australia; 9 The George Institute for Global Health, Imperial College, London, United Kingdom; 10 Fresenius Medical Care Deutschland GmbH, Global Medical Office, Bad Homburg v.d.H, Germany; 11 B. Braun Avitum AG, Medical Scientific Affairs, Melsungen, Germany; 12 Department of Nephrology, University College of London, London, United Kingdom; 13 Division of Nephrology, Department of Clinical Sciences, Lund University, Lund, Sweden; 14 Department of Nephrology, Aristotle University, Hippokration Hospital, Thessaloniki, Greece; 15 Department of Clinical Research and Epidemiology, Comprehensive Heart Failure Center, Würzburg, Germany; 16 Nuffield Department of Population Health, University of Oxford, Oxford, United Kingdom; 17 DaVita International, London, United Kingdom; 18 David Geffen School of Medicine, University of California Los Angeles, California, Los Angeles, United States of America; 19 Department Nephrologie-Hypertension Arterielle, Dialyses, Transplantation Renale, Tours, France; 20 Diaverum Renal Services Group, Budapest, Hungary; Faculty of Medicine, Saint-Joseph University, LEBANON

## Abstract

**Background:**

Frequent hemodialysis provided more than three times per week may lower mortality and improve health-related quality of life. Yet, the evidence is inconclusive. We evaluated the benefits and harms of frequent hemodialysis in people with kidney failure compared with standard hemodialysis.

**Methods:**

We performed a systematic review of randomized controlled trials including adults on hemodialysis with highly sensitive searching in MEDLINE, Embase, CENTRAL, and Google Scholar on 3 January 2024. Data were pooled using random-effects meta-analysis. Risk of bias was assessed using the Cochrane Risk of Bias 2 tool. We adjudicated evidence certainty using GRADE.

**Results:**

From 11,142 unique citations, only seven studies involving 518 participants proved eligible. The effects of frequent hemodialysis on physical and mental health were imprecise due to few data. Frequent hemodialysis probably had uncertain effect on death from all cause compared with standard hemodialysis (relative risk 0.79, 95% confidence interval 0.33–1.91, low certainty evidence). Data were not reported for death from cardiovascular causes, major cardiovascular events, fatigue or vascular access.

**Conclusion:**

The evidentiary basis for frequent hemodialysis is incomplete due to clinical trials with few or no events reported for mortality and cardiovascular outcome measures and few participants in which patient-reported outcomes including health-related quality of life and symptoms were reported.

## Introduction

Kidney failure is the tenth most frequent cause of death and is estimated to become the fifth leading cause of premature death by 2040 [1]. Kidney failure requires kidney replacement therapy (KRT), including dialysis and kidney transplantation, and is associated with high morbidity, mortality, and excess health-care costs [2]. Hemodialysis is used for 90% of patients when the kidney function falls below 10% of normal in healthcare settings with access to dialysis care [3]. However, major cardiovascular events, infections, hospital admissions and impaired quality of life are severe and frequent among patients treated with hemodialysis [4]. Standard thrice-weekly hemodialysis is still associated with severe fatigue, shortness of breath, low appetite, and bodily itch [5].

The high rates of death and morbidity among people treated with standard hemodialysis for kidney failure motivates an ongoing search for effective dialysis interventions, including convective therapies, medium cut-off membrane hemodialysis, and frequent or longer hours hemodialysis. Previous randomized and observational studies have shown that frequent dialysis, conducted more often than three times a week, provides more effective removal of fluid and uremic toxins, improves cardiovascular surrogates and quality of life, but the effect on survival in individual studies has remained uncertain [6, 7]. Accordingly, frequent hemodialysis has limited uptake as since 1950 three times a week has been set as the standard. Furthermore, reimbursement of dialysis treatments is based on the standard three times weekly dialysis schedule [8].

Given the uncertain evidence for frequent hemodialysis (4 or more sessions per week), we conducted a systematic review to evaluate the benefits and harms of frequent hemodialysis compared with standard hemodialysis in people with kidney failure.

## Materials and methods

### Study design

We pre-published the protocol (https://doi.org/10.17605/OSF.IO/3HVX5). The study was reported according to the Preferred Reporting Items for Systematic Reviews and Meta-analyses (PRISMA) guidelines for systematic reviews [9].

### Search strategy and selection criteria

A highly sensitive search strategy was conducted in MEDLINE, Embase and the Cochrane Central Register of Controlled Trials (CENTRAL), and the grey literature has been searched using Google Scholar from inception to 3 January 2024 without language restrictions (S1 File). The search keywords comprised “hemodialysis”; “hemodiafiltration”; “chronic kidney disease”; “kidney failure”; “end-stage kidney disease”; “frequency”; “extended”; “duration”; and “randomized controlled trial”; however, the full list of relevant keywords has been reported in the S1 File. Randomized controlled trials were eligible whether they evaluated frequent hemodialysis for treatment of kidney failure in adults on hemodialysis for at least 3 months. Frequent hemodialysis was defined as any hemodialysis schedule occurring more than three times a week, regardless of the duration of each hemodialysis session. Standard hemodialysis was defined as three times a week. We included studies of any duration. Quasi-RCTs (such as those studies in which treatment allocation was performed by alternation, use of alternate medical records, or days of the week) were excluded. Crossover studies and cluster randomized controlled trials were eligible. Two trained reviewers (PN and SG) screened the title and abstracts of retrieved citations to identify potentially eligible trials. Citation screening was conducted in Covidence [10]. Full texts were reviewed by PN and SG and eligible studies were included.

### Data extraction

Two trained reviewers (PN and SG) extracted data from each eligible study using a peer-reviewed standardized extraction form in Covidence. Data extraction included study characteristics (year of publication, country, type of study design, duration, funding, trial registration number), participant characteristics (setting, sample size, age, sex, years on dialysis, comorbidities), description of the intervention (type of hemodialysis, frequency, hours per session), and outcomes. For studies published in multiple articles, we included data from all sources, and extracted the most complete data and the longest follow-up. Data from trial registries were also extracted when available. The primary review outcome was health-related quality of life (including physical or mental health). The secondary outcomes were death from all causes, death from cardiovascular causes, major adverse cardiovascular events, dialysis vascular access interventions, fatigue, serious adverse events, and adverse events of special interest and included the highest priority outcomes identified by the Standardized Outcomes in Nephrology Haemodialysis initiative [11]. We planned to report the following outcomes in the Summary of Findings (SoF) Table: physical health, mental health, death from all causes, death from cardiovascular causes, major cardiovascular events, fatigue, and dialysis vascular access interventions. No unpublished studies have been identified. Missing data were handled either contacting authors or calculation mean and standsrd deviations from the row data as possible. No further data have been obtained from other sources.

### Risks of bias and quality assessment

Two reviewers (PN and SG) assessed the risk of bias in the included studies using the Cochrane Risk of Bias tool [12]. The Grading of Recommendations, Assessment, Development and Evaluation (GRADE) approach was used to appraise the certainty of evidence, assessed as high, moderate, low, or very low certainty evidence [13].

### Statistical analysis

Treatment effects were estimated by random-effects pairwise meta-analysis. We estimated effects as a relative risk (RR) for binary outcomes, mean difference (MD) for continuous outcomes reported on the same scale, or standardized mean difference (SMD) when continuous outcomes were reported on different scales, together with corresponding 95% confidence intervals (CI). SMDs were estimated by Hedges’ g [14]. Studies analyzing change scores were reported separately in meta-analyses from those reporting endpoint outcome data. Heterogeneity was first assessed with visual inspection of the forest plot. The I^2^-statistic was also used to quantify the percentage of variation across the studies that was due to heterogeneity. We used the following guide for interpretation of I^2^ values: 0% to 39%: not important heterogeneity; 40 to 79%: moderate heterogeneity; and 80% to 100%: substantial heterogeneity. We examined for evidence of small study effects by assessment of asymmetry in funnel plots if at least 10 studies were reported for the selected outcome.

### Subgroup and sensitivity analyses

Subgroup analyses to explore potential sources of heterogeneity by population characteristics (age 65 years or older and younger than 65 years, and diabetes), and setting of hemodialysis (home and facility) and sensitivity analyses (excluding unpublished studies, taking into account the risk of bias, excluding large studies to establish how much they dominate the results) were not performed.

## Results

**Fig 1:**
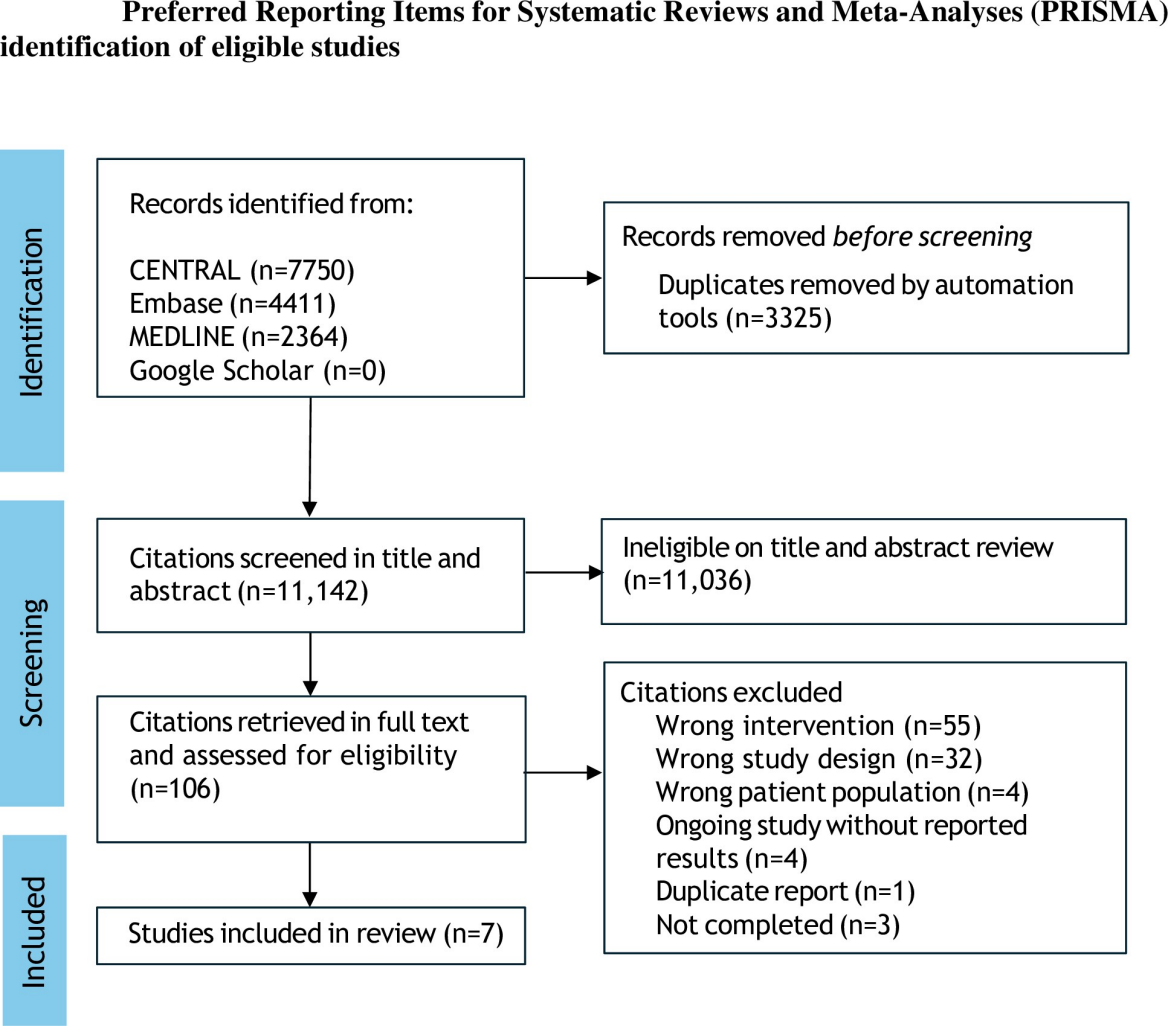
Flow chart.

The electronic search retrieved 11,142 unique citations (Fig 1). The full list of citations and reasons for exclusion are available in COVIDENCE at https://app.covidence.org/reviews/336588. Overall, seven trials involving 518 participants proved eligible for inclusion (S1 File). Publication ranged from 2001 to 2022. The trial mean age ranged from 48.9 to 64.1 years, and the proportion of men ranged from 33.3% to 69%. The median trial follow-up duration was 6 months. Frequent hemodialysis was prescribed between five and seven days a week, with variable session duration. Dialysis vintage was inconsistently reported. Four studies received support from government only and in the remaining studies there was no information on the source of funding. Trial inclusion criteria and dialysis prescriptions are described in the S1 File. Three studies were parallel group and four studies were crossover design. The individual study risk of bias is shown in the S1 File. Four studies were adjudicated as at low risk of bias overall, with one study identified as having some concerns related to risk of bias, due to the period or carry-over effects, measurement of outcomes, and selection of the reported results.

### Outcomes

Two trials including 332 participants reported the primary review outcomes of physical health and mental health (Tables 1 and 2 and in the S1 File). Instruments used to measure physical health and mental health were reported in the S1 File.

**Table 1:** Summary of findings.

Outcome	No. of studies reporting outcome	Frequent hemodialysis	Standard hemodialysis	Relative Risk or Mean Difference	I^2^	Certainty of the evidence (GRADE)
Physical health	2	138	129	MD -0.02 (-0.48, 0.44)	67.19	Low
Mental health	2	135	127	MD 0.25 (0.01, 0.49)	0.00	Low
Death from any cause	5	234	227	RR 0.79 (0.33, 1.91)	0.00	Low
Death from cardiovascular causes	2	39	40	RR 1.05 (0.07, 16.04)	0.00	Very low
Major cardiovascular events	-	-	-	-	-	-
Fatigue	-	-	-	-	-	-
Dialysis vascular access interventions	-	-	-	-	-	-

MD: mean difference; RR: relative risk. Note: physical and mental health were related to physical or mental component of quality of life. GRADE Working Group grades of evidence. High certainty: we are very confident that the true effect lies close to that of the estimate of the effect. Moderate certainty: we are moderately confident in the effect estimate: the true effect is likely to be close to the estimate of the effect, but there is a possibility that is substantially different. Low certainty: our confidence in the effect estimate is limited: the true effect may be substantially different from the estimate of the effect. Very low certainty: we have little confidence in the effect estimate: the true effect is likely to be substantially different from the estimate of the effect.

**Table 2:** Physical health and mental health outcomes.

	Time of assessment	N of participants				Results		
Study		Frequency HD	Standard HD	Instrument	Outcome measure	Frequent	Standard	Notes
Fagugli 2001 [15]	-	-	-	-	-	-	-	-
Culleton 2007 [16]	Baseline 6 months	22	22	EQ-5D index	Change (6 mo)	0.05 (–0.07, 0.17)*		Frequent nocturnal HD did not improve the change in EQ-5D index scores from baseline.
				KDQOL	Change (6 mo)			
				Symptom/Problems	Change (6 mo)	Graphical display		
				Effects of kidney disease	Change (6 mo)	Graphical display		
					Change (6 mo)	Graphical display		
								Frequent nocturnal HD improved the domains of “effects of kidney disease” and “burden of kidney disease”.
				Burden of kidney disease	Change (6 mo)	Graphical display		
				Sleep				
FHN Trial 2010 [17]	Baseline	96	81	Short physical performance battery score	Mean (12 mo)	8.4±2.8	7.9±2.8	Improvement in self-reported physical functioning with frequent HD that did not reach statistical significance.
	4 months				Unadjusted change (12 mo)	0.25±0.30		
	12 months				Adjusted change (12 mo)	–0.20±0.19	–0.41±0.21	
					Adjusted treatment effect (12 mo)	0.21 (–0.34, 0.76)		
		100	90	Physical health composite (RAND-36)	Mean (12 mo)	42.1±10.8	38.6±9.5	
					Unadjusted change (12 mo)	3.3±1.3		
					Adjusted change (12 mo)	3.4±0.8	0.4±0.8	
					Adjusted treatment effect (12 mo)	2.9 (0.8, 5.1)		
		102	90	Physical functioning (RAND-36)	Mean (12 mo)	64.0±27.7	59.1±24.7	
					Unadjusted change (12 mo)	6.5±3.3		
					Adjusted change (12 mo)	4.5±2.1	0.0±2.2	
					Adjusted treatment effect (12 mo)	4.4 (–1.3, 10.2)		
FHN Nocturnal 2011 [18]	Baseline	34	37	Short physical performance battery score	Mean (12 mo)	7.8±3.4	8.7±2.8	No significant different between groups in self-reported physical functioning.
	4 months				Unadjusted change (12 mo)	0.35±0.61		
	12 months				Adjusted change (12 mo)			
					Adjusted treatment effect (12 mo)	–0.92±0.44	–0.41±0.43	
						–0.50 (–1.71, 0.70)		

		36	38	Physical health composite (RAND-36)	Mean (12 mo)	39.8±12.2	40.6±9.2	
					Unadjusted change (12 mo)	1.2±2.1		
					Adjusted change (12 mo)	2.7±1.4	2.1±1.5	
					Adjusted treatment effect (12 mo)	0.6 (–3.4, 4.7)		
		36	39	Physical functioning (RAND-36)	Mean (12 mo)	55.0±34.3	63.5±23.4	
					Unadjusted change (12 mo)	–3.7±5.0		
					Adjusted change (12 mo)	–3.1±3.5	1.1±3.6	
					Adjusted treatment effect (12 mo)	–4.2 (–14.1, 5.7)		
		39	38	BDI	Mean (12 mo)	9.7±8.6	11.1±10.2	
					Unadjusted change (12 mo)			
					Adjusted change (12 mo)	–2.1±5.2	–0.6±9.6	
					Adjusted treatment effect (12 mo)			
						–1.9±1.2	–0.4±1.3	
						–1.5 (–4.9, 1.9)		
Di Micco 2012 [19]	-	-	-	-	-	-	-	-
Zimmerman 2014 [20]	-	-	-	-	-	-	-	-
Moya 2022 [21]	-	-	-	-	-	-	-	-

*This data was not reported in a proper format to be included in the meta-analysis. Data were reported as mean difference or standardized mean difference and 95% confidence interval (CI). N: number. Mo: months. EQ-5D index: EuroQol 5-Dialysis; KDQOL: Kidney Disease Quality of Life; BDI: Beck Depression Inventory; RAND-36: RAND-36 Item Health Survey.

Due to differences in measurement tools and imprecision in the results, the evidence for effects of frequent hemodialysis on physical health was of low certainty. Data for mental health, depression, and sleep quality were limited. There was no evidence that, compared to standard hemodialysis, frequent hemodialysis had any clinically important effects on physical or mental health. Frequent hemodialysis may have uncertain effects on death from all cause compared with standard hemodialysis (relative risk (RR) 0.79, 95% confidence interval (CI) 0.33–1.91, low certainty evidence) (S1 File), or need to access intervention. Data were not available for the outcomes of death from cardiovascular causes, or major cardiovascular events, including myocardial infarction, stroke, or peripheral arterial events.

## Discussion

Our review showed that the evidence of the effects of frequent hemodialysis in people with kidney failure is still limited. The current state of the evidence based on RCTs with short follow-up does not support neither refutes that frequent dialysis beneficially affects or harms the outcomes, such as quality of life, physical or mental health compared with standard hemodialysis. Frequent hemodialysis may have uncertain effect on death from all cause compared with standard hemodialysis, and limited or no events were reported for death from cardiovascular causes, need to access intervention, major cardiovascular events (e.g., myocardial infarction, stroke, or peripheral arterial events), fatigue and vascular access. Data for depression and sleep quality were sparse. Adverse events were inconsistently reported. Overall, clinical trials used different measures or reported imprecision in data, leading to low or very low evidence certainty.

For our knowledge this is the first systematic review assessing the efficacy and safety of frequent hemodialysis compared with standard hemodialysis for the treatment of kidney failure in RCTs only. A previous review that included both primary studies and systematic reviews evaluated the effects of frequent hemodialysis on surrogate outcomes, showing that frequent dialysis improved blood pressure management that may potentially lead to improvement in survival, cardiovascular outcomes, and quality of life, although no outcome data were reported to support these results [22]. Our findings partly agreed with data reported in other studies based on randomized and observational studies, that showed that nocturnal hemodialysis did not have any difference in mortality, but improved quality of life compared to standard hemodialysis and this evaluation could overestimate the results due to methodological issue in the study selection [23, 24].

This review was performed using a highly sensitive search strategy including all available clinical trials. Data extraction and analysis were performed by two independent reviewers, who used the risk of bias Cochrane tool and the GRADE approach to assess the quality of the evidence. However, this study reported some limitations due to the individual studies that should be considered for interpreting our findings. Firstly, the majority of the eligible studies were not designed to report key clinical or patient-reported outcomes that could be underestimated or underreported, and cardiovascular adverse events were not reported. Secondly, clinical trials were designed to estimate the effects of surrogate outcomes, or follow-up was insufficient to address critical outcome data (including death and cardiovascular outcomes) or clearly assess whether more frequent dialysis sessions may lead to premature loss of residual renal function. Thirdly, the studies had poor methodological quality that may have reduced our certainty in the estimated treatment effects, due to the imprecision in the estimates in the small studies with short follow-up duration. Lastly, heterogeneity and publication bias could be not explored due to the limited number of studies or events.

This systematic review has shown that the effects of frequent hemodialysis compared to standard haemodialysis are uncertain on mortality, cardiovascular events, and quality of life. The benefit and harms of treatment in kidney failure are insufficient to inform practice in adults on hemodialysis. The current evidence appears to be a lower research priority and is unable to guide treatment decision-making. Future high quality and well powered RCTs should be designed and conducted to evaluate the efficacy of frequent versus standard hemodialysis for the treatment of kidney failure in long-term. It is likely that future studies could change the estimated effects of treatments for frequent or standard dialysis and should evaluate outcomes prioritized by stakeholders, including overall health-related quality of life or more specific patient-reported outcomes, such as dialysis symptoms or fatigue [25], followed by evaluation of cost for treatments in kidney failure to inform clinical practice.

## Supporting information

S1 File (DOCX)

S1 Checklist PRISMA 2020 checklist. (DOCX)
