# Correction: Frequent hemodialysis versus standard hemodialysis for people with kidney failure: Systematic review and meta-analysis of randomized controlled trials

**DOI:** 10.1371/journal.pone.0332338

**Published:** 2025-09-15

**Authors:** Patrizia Natale, Suetonia C. Green, Matthias Rose, Michiel L. Bots, Peter J. Blankestijn, Robin W. M. Vernooij, Karin G. F. Gerritsen, Mark Woodward, Carinna Hockham, Krister Cromm, Claudia Barth, Andrew Davenport, Jörgen Hegbrant, Pantelis Sarafidis, Partha Das, Christoph Wanner, Allan R. Nissenson, Benedicte Sautenet, Marietta Török, Giovanni Strippoli

The fourth author’s name is spelled incorrectly. The correct name is: Karin G.F. Gerritsen.
