# Supplementary material for: Frequent hemodialysis versus standard hemodialysis for people with kidney failure: Systematic review and meta-analysis of randomized controlled trials
Source: PLoS One. 2024 Sep 6;19(9):e0309773. doi: 10.1371/journal.pone.0309773 (PMC11379176; doi:10.1371/journal.pone.0309773)
Supplement: S1 File — (DOCX) [file pone.0309773.s001.docx]

# Supplementary Materials

## S1 Table. Search Strategy

CENTRAL with the inception on the 3^rd^ January 2024

1. MeSH descriptor Renal Dialysis explode all trees
2. MeSH descriptor Hemofiltration explode all trees
3. MeSH descriptor Kidney Failure, Chronic, this term only
4. (hemodialysis):ti,ab,kw or (haemodialysis):ti,ab,kw or (dialysis):ti,ab,kw in Trials
5. (hemofiltration or haemofiltration):ti,ab,kw in Trials
6. (hemodiafiltration or haemodiafiltration):ti,ab,kw in Trials
7. (end‐stage kidney or end‐stage renal or endstage kidney or endstage renal):ti,ab,kw in Trials
8. (ESKD or ESKF or ESRD or ESRF):ti,ab,kw in Trials
9. (eskd or eskf or esrd or esrf):ti,ab,kw in Trials
10. #1 or #2 or #3 or #4 or #5 or #6 or $7 or #8 or #9
11. (duration):ti,ab,kw in Trials
12. (daily):ti,ab,kw in Trials
13. (frequen*):ti,ab,kw in Trials
14. (extended):ti,ab,kw in Trials
15. (intensi*):ti,ab,kw in Trials
16. (hour*):ti,ab,kw in Trials
17. #11 or #12 or #13 or #14 or #15 or #16
18. #10 and #17

MEDLINE (OVID) with the inception on the 3^rd^ January 2024

1. exp Renal Dialysis/
2. exp Hemofiltration/
3. Kidney Failure, Chronic/
4. dialysis.tw.
5. (hemodialysis or haemodialysis).tw.
6. (hemofiltration or haemofiltration).tw.
7. (hemodiafiltration or haemodiafiltration).tw.
8. (end‐stage kidney or end‐stage renal or endstage kidney or endstage renal).tw.
9. (ESKD OR ESKF OR ESRD OR ESRF).tw.
10. Or/1-9
11. duration.tw.
12. (short adj2 daily).tw.
13. frequen$.tw.
14. extend$.tw.
15. adequacy.tw.
16. initiat$.tw.
17. intensi$.tw.
18. or/1-17
19. randomized controlled trial.pt.
20. controlled clinical trial.pt.
21. randomized.ab.
22. randomly.ab.
23. trial.ab.
24. or/19-23
25. exp animals/ not humans.sh.
26. 24 not 25
27. 10 and 18 and 26

EMBASE (OVID) with the inception on the 3^rd^ January 2024

1. exp Renal Replacement Therapy/
2. (hemodialysis or haemodialysis).tw.
3. (hemofiltration or haemofiltration).tw.
4. (hemodiafiltration or haemodiafiltration).tw.
5. dialysis.tw.
6. Chronic Kidney Disease/
7. Kidney Failure/
8. Chronic Kidney Failure/
9. (end*stage renal or end*stage kidney or endstage renal or endstage kidney).tw.
10. (ESRF or ESKF or ESRD or ESKD).tw.
11. Or/1-10
12. duration.tw.
13. (short adj2 daily).tw.
14. frequen$.tw.
15. extended.tw.
16. extend$.tw.
17. intensi$.tw.
18. or/12-17
19. random$.tw.
20. (doubl$ adj blind$).tw.
21. factorial$.tw.
22. allocate$.tw.
23. randomization.sh.
24. trial$.tw.
25. randomized controlled trial.sh.
26. assign$.tw.
27. or/19-26
28. 11 and 18 and 27

## S2 Table. Trial characteristics

| **Author, Year, Reference** | **Trial design** | **Treatment group** | **N** | **Duration on dialysis** | **Mean age (years)** | **Men, (%)** | **Diabetes, %** | **Cardio**  **vascular disease, %** | **Fistula, %** | **Intervention** | **Inclusion criteria** | **Follow-up** |
| --- | --- | --- | --- | --- | --- | --- | --- | --- | --- | --- | --- | --- |
| Fagugli 2001 (1) | Cross-over | Daily hemodialysis | 12 | 1218 days | 64.1 | 33.3 | 25.0 | - | - | Daily hemodialysis (12 hours per week) | Hypertension and kidney failure previously treated with hemodialysis for at least 6 months | 6 months |
|  |  | Standard hemodialysis |  |  |  |  |  |  |  | Three times per week (12 hours per week) |  |  |
| Culleton 2007 (2) | Parallel | Nocturnal hemodialysis | 27 | 5.5 years | 55.1 | 69 | 38 | 38 | 58 | Nocturnal hemodialysis 5–6 nights per week for minimum of 6 hours | 18 years old, receiving self-care or conventional home hemodialysis 3 times weekly | 6 months |
|  |  | Standard hemodialysis | 25 | 4.8 years | 53.1 | 56 | 44 | 40 | 56 | Three times weekly hemodialysis with single-pool Kt/V >1.2 |  |  |
| FHN Trial 2010 (3) | Parallel | Frequent hemodialysis | 125 | - | 48.9 | 62.4 | 40.0 | 8.8 | 65.6 | Six times per week, 1.5–2.75 hours, targeted equilibrated Kt/V_urea_ 0.9 | Kidney failure, age ≥ 13 years, achieved eKt/V ≥ 1.0 for the last two baseline hemodialysis sessions, weight >30 kg | 12 months |
|  |  | Standard hemodialysis | 120 | - | 52.0 | 60.8 | 41.7 | 13.3 | 62.5 | Three times per week, 2.5–4.0 hours, targeted equilibrated Kt/V_urea_ 1.1 |  |  |
| FHN Nocturnal Trial 2011 (4) | Parallel | Frequent nocturnal hemodialysis | 45 | - | 51.7 | 64.4 | 42.2 | 11.1 | 53.3 | Six times a week for ≥6 hours per session | Kidney failure ≥ 18 years of age, achieved mean eKt/V≥1.0 for last 2 baseline hemodialysis sessions, willing to perform hemodialysis at home | 12 months |
|  |  | Standard hemodialysis | 42 | - | 52.8 | 66.7 | 42.9 | 9.5 | 40.5 | Three times a week for <5 hours per session |  |  |
| Di Micco 2012 (5) | Cross-over | Daily hemodialysis | 30 | - | - | - | - | - | - | Daily dialysis, 180 min/session | Kidney failure with a urinary output <200 mL per day, treatment with chronic standard bicarbonate hemodialysis for at least 3 months and a steady dry weight achieved in the previous 3 months | 1 week |
|  |  | Standard hemodialysis | 30 | - | - | - | - | - | - | Three times per week, 240 min/session |  |  |
| Zimmerman 2014 (6) | Cross-over | Short daily hemodialysis | 22 | 1368 days | 53 | 68.4 | 57.9 | - | 57.9 | Hemodialysis 6 times per week with 50% reduction in session length | Hemodialysis >3 months, 1 or 2 anti-hypertensives with pre-dialysis systolic blood pressure >140 mmHg over 1 month, or 3 or more anti-hypertensive regardless of pre-dialysis systolic pressure, able to commit to daily therapy | 3 months |
|  |  | Standard hemodialysis |  |  |  |  |  |  |  | Hemodialysis 3 times per week |  |  |
| Moya 2022 (7) | Cross-over | Frequent hemodialysis | 40 | - | - | - | - | - | - | Short, frequent 5 days per week, 2.5 hours per session using portable machine | Adults with kidney failure treated with hemodialysis | … |
|  |  | Standard hemodialysis |  | - | - | - | - | - | - | Three times per week in-centre online hemodiafiltration, 4 hours er session |  |  |

## S3 Table. Risk of bias in eligible studies

##

## S4 Table. Summary table of instruments used to measure physical health and mental health

| **Instrument** | **Description** | **No. of questions** | **Domains** | **Range** | **Minimal Clinically Important Difference** |
| --- | --- | --- | --- | --- | --- |
| BDI (8) | Depressive symptoms | 21 items | Affective (items ‘‘sadness’’, ‘‘loss of pleasure’’, ‘‘crying’’, and ‘‘indecisiveness’’)  Motivational (items ‘‘pessimism’’ and ‘‘suicidal thoughts’’)  Cognitive’’ (items ‘‘loss of interest’’ and ‘‘concentration difficulty’’)  Cognitive distortions’ (items ‘‘past failure’’, ‘‘guilty feelings’’, ‘‘punishment feelings’’, ‘‘self-dislike’’, ‘‘self-criticalness’’, and ‘‘worthlessness’’)  Behavioral’ (items ‘‘agitation’’, ‘‘loss of energy’’, ‘‘irritability’’, and ‘‘tiredness or fatigue’’)  Vegetative’(items ‘‘changes in sleep’’, ‘‘changes in appetite’’, and ‘‘loss of interest in sex’’) | 1-10 normal  11-16 Mild mood disturbance  17-20 Borderline clinical depression  21-30 Moderate depression  31-40 Severe depression  Over 40 Extreme depression | 17.5% reduction in scores (9) |
| EQ-5D (10) | Disease-specific | 6 | 5 dimensions:  Mobility  Self-care  Usual activities  Pain/discomfort  Anxiety/depression | No problems  Slight problems  Moderate problems  Severe problems  Extreme problem  0–100 on VAS although depends on utility function (may have scores as “worse than dead”) | 0.03–0.05 (11) |
| KDQOL (12) | Health survey and additional multi-item kidney disease-targeted scales and an overall health rating item | 36 items | Symptoms/problems  Effects of kidney disease  Burden of kidney disease  Work status  Cognitive function  Quality of social interaction  Sexual function  Sleep  Social support  Dialysis staff encouragement  Patient satisfaction | 0–100 | 5 (13) |
| RAND-36 (14) | General health survey | 36 items | 8 domains  Physical functioning  Role physical  Pain index  General health items  Energy/fatigue  Social functioning  Role emotional  Emotional wellbeing  Health transition | 0–100 | 3–5 (15) |
| Short physical performance battery score (16) | Assessed physical performance | 3 tests | Three domains  Balance  Strength  Gait measurements | 0–12 | 3 points (12) |

EQ-5D index: EuroQol 5-Dialysis; KDQOL: Kidney Disease Quality of Life; BDI: Beck Depression Inventory; RAND-36: RAND-36 Item Health Survey.

**S1 Figure. Forest plot of treatment effects of physical health**

**
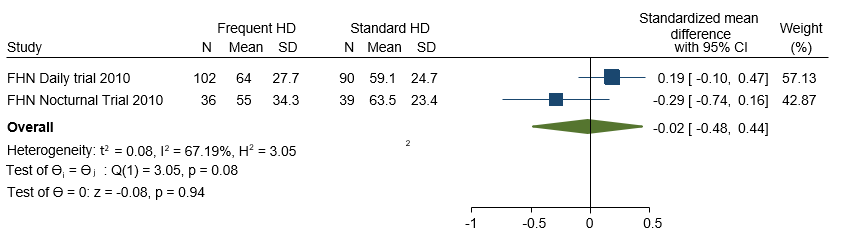
**

**S2 Figure. Forest plot of treatment effects of mental health**

**
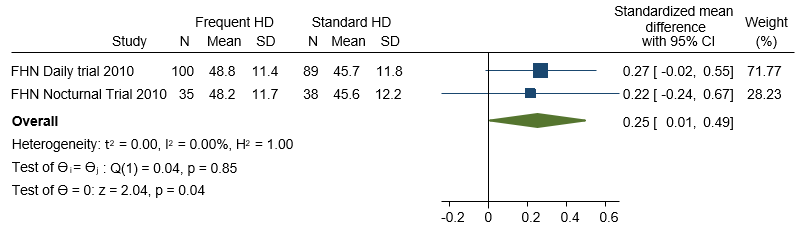
**

**S3 Figure. Forest plot of treatment effects on death from any cause**

**
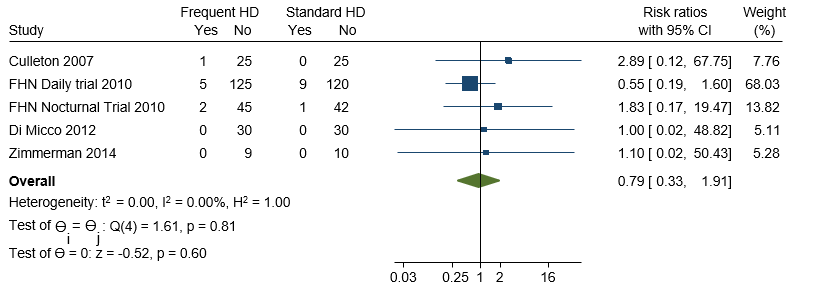
**

**S4 Figure. Forest plot of treatment effects of death from cardiovascular causes**

**
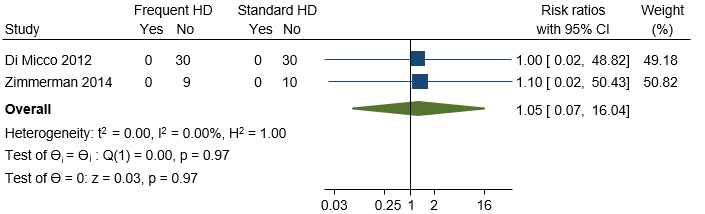
**

**S5 Figure. Forest plot of treatment effects of need to access intervention**

**
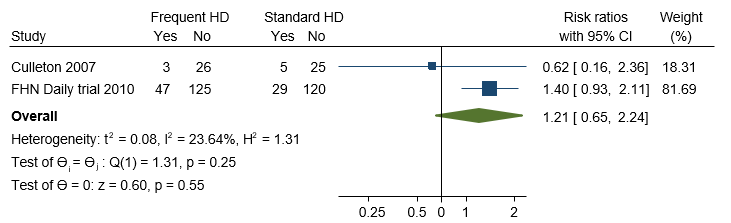
**

**S6 Figure. Forest plot of treatment effects of depression**

**
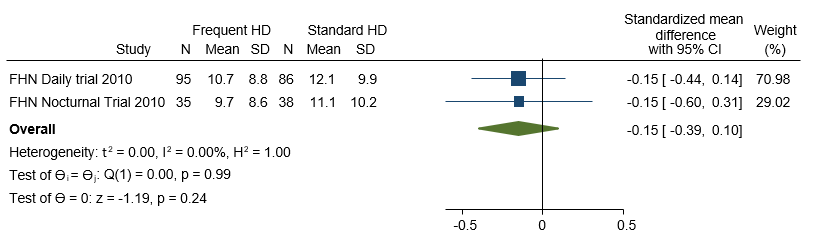
**

**S7 Figure. Forest plot of treatment effects of sleep**

**
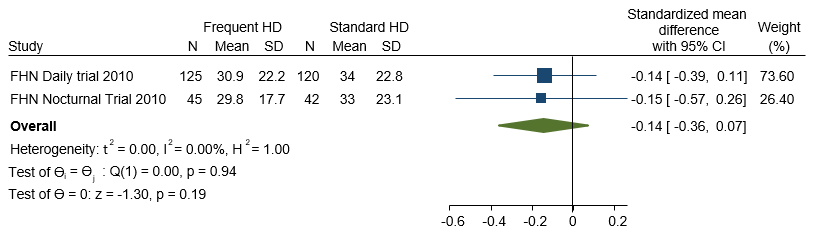
**

## References

1. Fagugli RM, Reboldi G, Quintaliani G, Pasini P, Ciao G, Cicconi B, et al. Short daily hemodialysis: Blood pressure control and left ventricular mass reduction in hypertensive hemodialysis patients. *Am J Kidney Dis*. 2001;38(2):371–6.

2. Culleton BF, Walsh M, Klarenbach SW, Mortis G, Scott-Douglas N, Quinn RR, et al. Effect of frequent nocturnal hemodialysis vs conventional hemodialysis on left ventricular mass and quality of life. *JAMA*. 2007;298(11):1291.

3. FHN Trial Group; Chertow GM, Levin NW, Beck GJ, Depner TA, Eggers PW, et al. In-center hemodialysis six times per week versus three times per week. *N Engl J Med*. 2010;363(24):2287–300.

4. Rocco MV, Lockridge RS, Beck GJ, Eggers PW, Gassman JJ, Greene T, et al.. The effects of frequent nocturnal home hemodialysis: the Frequent Hemodialysis Network Nocturnal Trial. *Kidney Int*. 2011;80(10):1080–91.

5. Di Micco L, Torraca S, Sirico ML, Tartaglia D, Di Iorio B. Daily dialysis reduces pulse wave velocity in chronic hemodialysis patients. *Hypertens Res*. 2012;35(5):518–22.

6. Zimmerman DL, Ruzicka M, Hebert P, Fergusson D, Touyz RM, Burns KD. Short daily versus conventional hemodialysis for hypertensive patients: A randomized cross-over study. *PLoS One*. 2014;9(5):e97135.

7. González Moya M, Molina P, Molina M, García-Valdelvira M, Vizcaíno B, Montesa Marín M, et al. Elimination of middle and large uraemic toxins in short daily home haemodialysis with low dialysate volume: A randomized crossover clinical trial. *Nephrol Dial Transplant*. 2022;37(Supplement 3).

8. Beck AT, Steer R, Brown GK. Beck depression inventory-II: Manual. *The Psychological Corporation, San Antonio, TX*. 1996.

9. Button KS, Kounali D, Thomas L, Wiles NJ, Peters TJ, Welton NJ, et al. Minimal clinically important difference on the Beck Depression Inventory--II according to the patient's perspective. *Psychol Med*. 2015;45(15):3269-79.

10. Rabin R, Charro FD. EQ-SD: a measure of health status from the EuroQol Group. *Ann Med*. 2001;33(5):337–43.

11. McClure NS, Sayah FA, Ohinmaa A, Johnson JA. Minimally Important Difference of the EQ-5D-5L Index Score in Adults with Type 2 Diabetes. *Value Health*. 2018;21(9):1090–7.

12. Hays RD, Kallich JD, Mapes DL, Coons SJ, Carter WB. Development of the kidney disease quality of life (KDQOL) instrument. *Qual Life Res*. 1994;5:329–38.

13. Jaeschke R, Singer J, Guyatt GH. Measurement of health status. *Control Clin Trials*. 1989;10(4):407–15.

14. Hays RD, Sherbourne C, Mazel RM. The RAND 36-Item Health Survey 1.0. *Health Econ*. 1993;2(3):217–27.

15. Samsa G, Edelman D, Rothman ML, Williams GR, Lipscomb J, Matchar D. Determining clinically important differences in health status measures: a general approach with illustration to the Health Utilities Index Mark II. *Pharmacoeconomics*. 1999;15(2):141–55.

16. Guralnik JM, Simonsick EM, Ferrucci L, Glynn RJ, Berkman LF, Blazer DG, et al. A short physical performance battery assessing lower extremity function: association with self-reported disability and prediction of mortality and nursing home admission. *J Gerontol*. 1994;49(2):M85–94.
